# The Case of the Missing Mechanism: How Does Temperature Influence Seasonal Timing in Endotherms?

**DOI:** 10.1371/journal.pbio.1001517

**Published:** 2013-04-02

**Authors:** Samuel P. Caro, Sonja V. Schaper, Roelof A. Hut, Gregory F. Ball, Marcel E. Visser

**Affiliations:** 1 Department of Animal Ecology, Netherlands Institute of Ecology (NIOO-KNAW), Wageningen, The Netherlands; 2 Chronobiology Unit, Center for Behavior and Neurosciences (CBN), University of Groningen, Groningen, The Netherlands; 3 Department of Psychological and Brain Sciences, Johns Hopkins University, Baltimore, Maryland, United States of America

## Abstract

Temperature has a strong effect on the seasonal timing of life-history stages in both mammals and birds, even though these species can regulate their body temperature under a wide range of ambient temperatures. Correlational studies showing this effect have recently been supported by experiments demonstrating a direct, causal relationship between ambient temperature and seasonal timing. Predicting how endotherms will respond to global warming requires an understanding of the physiological mechanisms by which temperature affects the seasonal timing of life histories. These mechanisms, however, remain obscure. We outline a road map for research aimed at identifying the pathways through which temperature is translated into seasonal timing.

## Abstract 2

How temperature affects the timing of life cycles in warm-blooded organisms remains a mystery but must be addressed in order to predict the future consequences of global warming.

## Introduction

Seasonal timing of life-cycle events like reproduction, migration, hibernation, and molt has major fitness consequences. In a given environment, the timing of the optimal periods for each of these stages varies from year to year, which leads to strong interannual variation in timing of the life-cycle stages in many species. While day length (photoperiod) influences seasonal timing [1],[2], it cannot account for interannual variation. Ambient temperature is the environmental variable that often best correlates with this variation in timing: many species flower, breed, or end hibernation earlier in warmer years [3]–[6].

Seasonal timing is correlated to temperatures, which have increased at an unprecedented rate over the past decades because of global warming. These increases have in turn led to changes in seasonal timing, and many species, including humans, are currently shifting their seasonal behaviors [7],[8]. However, plants, insects, and vertebrates shift their timing differently, possibly because the relevant temperatures for these groups change at different rates, or because (the nonchanging) photoperiod plays a more important role in timing in some groups than in others. As a consequence, many organisms become progressively mismatched to their food supply [9]. This increasing mismatch in timing can affect population viability and lead to natural selection on the mechanisms underlying timing, especially on the intensity with which ambient temperature affects timing. The key question is whether species will be able to adapt fast enough to keep up with their changing world [10].

A large body of literature, using excellent phenological time-series and large spatiotemporal datasets, demonstrates a correlation between temperature and timing [3]–[6]. However, the temperature ranges as well as the seasonal temperature patterns under which these long-term datasets were collected have changed and will continue to change due to global warming. Therefore, historical data will not accurately predict how organisms will respond to these new conditions [10], highlighting the need for a more mechanistic understanding of how temperature affects seasonal timing [11]–[13]. We know that seasonal timing in animals is orchestrated by underlying neuroendocrine mechanisms, but for most taxa we don't understand how these mechanisms are affected by ambient temperature—a critical piece of the puzzle needed to predict how organisms might be constrained in their ability to change their timing under global warming [14]–[17].

Endotherms have evolved a unique system for maintaining a relatively constant body temperature under a large range of ambient temperatures. Nonetheless, most endothermic species living outside the tropics are still truly seasonal, with the most energy-demanding stages of their life cycles restricted to periods when temperature is mild, or when food (also often temperature-restricted) is plentiful [18]. While it is easy to conceive that temperature should affect seasonal timing in ectotherms, as ambient temperature directly limits enzymatic activities in these species, metabolism can function independently of ambient temperature in birds and mammals. This raises the central question: what physiological mechanisms operate in endotherms that allow ambient temperature to influence seasonal timing? We combine a mechanistic approach of temperature perception with insights from both natural observations and experimental studies about causal temperature effects on seasonal timing in endotherms to provide an integrated research road map to address this mystery.

## Does Temperature Directly Affect Seasonal Timing?

For most temperate-zone endotherms, the timing of many life-cycle stages correlates with ambient temperature: in warmer years, birds return from migration earlier, red deer give birth earlier, and marmots end hibernation and wean sooner [6],[19]–[21]. These correlations however do not necessarily mean that animals use temperature as a predictive cue for future environmental conditions. The apparent influence of temperature on timing may represent an energetic constraint or temperature may act via an indirect signaling cue, like vegetation development, for example. In both cases, animals would not need to directly sense ambient temperature to organize their life cycles, and we would not need to understand how temperature is perceived and integrated at the mechanistic level to predict the consequences of a warming climate. Thus, the first critical question is whether temperature has a direct signaling effect on seasonal timing in endotherms.

Demonstrating a causal effect of temperature on timing requires experiments under controlled conditions in the laboratory [22]. In birds, the first evidence for a direct relationship between ambient temperature and timing of reproduction has only recently been demonstrated (Box 1; [23],[24]). In mammals it has, to our knowledge, not yet been experimentally shown that temperature influences timing of breeding per se, but there is evidence that temperature modulates photoperiodic effects on the reproductive system [25]–[27]. Temperature has also been shown to regulate other life-cycle stages, like migration in birds [28] or hibernation in mammals (Box 2). Not surprisingly, it is not always the same temperature characteristic that affects these different stages. While the pattern of temperature increase affects breeding time (Box 1), it is absolute temperature that affects molt, migration, and hibernation (Box 2, [28],[29]): keeping weasels (*Mustela erminea bangsi*) or mice (*Peromyscus leucopus*) at high constant temperatures makes their summer pelage persist longer [30],[31], and makes hamsters (*Phodopus sungorus*) maintain high testis weight [32]. Altogether, these few first experimental studies help fill an important gap in our knowledge from correlational evidence observed in the wild to proximate temperature-mediating mechanisms. Given the scarcity of experimental approaches investigating this causal effect of temperature, especially in mammals, generalizations are not possible and additional studies are desperately needed.

**Box 1. Effects of temperature on timing of breeding in great tits**

The great tit (*Parus major*) is one of the most commonly studied songbird species. In the Netherlands, monitoring of the ecology of this species started at the beginning of the twentieth century, and an extensive amount of data has been accumulated on its breeding phenology. This includes studies on the correlational relationship between temperature and timing of breeding in the wild: in warmer years, Dutch great tits breed earlier [76]; and studies show that great tits advance their breeding phenology in response to global warming [77],[78]. In 1999, a series of experiments aimed at deciphering the possible causal relationship between temperature and breeding phenology were started. This research program made use of up to 36 climate-controlled aviaries in which single pairs of great tits were housed. During the first 6 years, birds were exposed to temperature patterns mimicking a particularly cold and a particularly warm spring (Figure IA) [79]. The average temperature difference between the treatments was only 4°C. Although year-to-year variation in the average laying dates was large despite the use of the same temperature patterns across years, a direct effect of temperature was demonstrated. In 5 out of 6 years, birds exposed to the warm treatment laid early compared to birds exposed to the cold treatment [79]. Once the causal relationship between temperature and laying dates was demonstrated, a second set of experiments aimed at identifying the characteristics of temperature that these birds use to time their breeding period was initiated. For this, artificial profiles of temperature were used. They varied either (i) continuously by 4°C over the spring, (ii) in the timing of a cold period, or (iii) in the onset and rate of increase during spring (Figure IB–D) [54],[80]. According to these experiments, it is essentially the periods during which temperature increases that play a role in the proximate determination of reproduction in the Dutch great tits; the absolute temperature value has no effect [80]. For example, birds exposed to temperatures increasing progressively during spring but constantly differing by 4°C (Figure IB) start breeding at the same time. However, a late spring increase in temperature will influence laying dates more than an early increase or a constant temperature (Figure IC–D) [80].

**Figure I:**
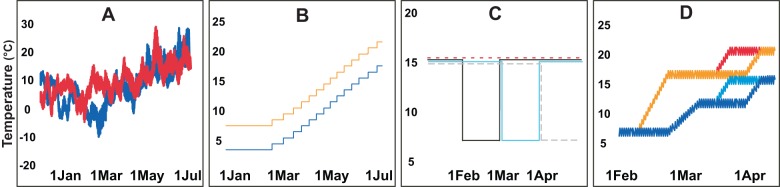
Temperature profiles used during experiments conducted in climate-controlled aviaries. A: Natural patterns of variation of temperature. B?D: Artificial patterns of temperature variation. B: Progressive increase of temperature with a constant 4uC difference. C: Variation in the onset and termination of a cold period. D: Variation in the onset and the rate of increase of temperature. Adapted from [54],[79],[80].

**Box 2. Effects of temperature on timing of hibernation in ground squirrels and other rodents**

Terrestrial mammals often rely on body reserves or food storages combined with metabolic suppression to overcome the harsh winter conditions. Several mammal species can be characterized as deep hibernators. In these animals, extreme metabolic suppression is found, with metabolic rates that can be reduced down to about 1% of normal euthermic resting metabolism [81] for about 5–9 months per year. Body temperature drops dramatically during that life-cycle stage, sometimes below freezing temperatures (as low as −2.9°C in the Arctic ground squirrel [82]). Hibernation is however generally not continuous and most hibernating species show intermittent interruptions of their low-temperature torpid states by short arousal phases that are modulated both by endogenous circannual cycles and by ambient temperatures [83],[84]. Due to this temperature dependency of arousal frequency, energy expenditure during hibernation is minimized at ambient temperatures around 0°C [85]. For most hibernators in temperate zones this means that increased winter temperatures will lead to increased energy expenditure, stronger decline in energy reserves, reduced winter survival, and possibly altered behavior and phenology. Indeed, hibernation energetics modeling in bats led to the prediction that the location of overwintering caves will be at higher latitudes as a response to global warming [85]. Hibernating species that do not migrate, such as squirrels, marmots, or hamsters, would rather have to adapt their hibernating phenology under global warming. Indeed, earlier snow melt and/or higher spring temperatures correlate with advanced hibernation end in ground squirrels [86],[87] and marmots [6]. Although this is expected to have detrimental effects on parental quality, the concomitant advancement in reproduction seems to be beneficial for juveniles that have more time to develop in preparation of the next winter [21]. The causality of these temperature effects on hibernation phenology was demonstrated in laboratory manipulations of ambient temperature during hibernation. In an experiment conducted in climate-controlled rooms, Nemeth et al. [88] showed that European ground squirrels maintained at 5°C and 9°C during their hibernation emerged significantly earlier in spring than squirrels maintained at 0°C (Figure IIA). In addition, squirrels exposed to the high temperature treatments did arouse more often and for longer periods of time, causing body mass to decrease more quickly than in the cold treatment [88]. In an earlier set of experiments with golden-mantled ground squirrels, it was shown that maintaining animals at 0°C advanced the onset of hibernation by about 40 days compared to animals kept at 21°C, even though food and water were constantly provided ad libitum [84] (Figure IIB). It was even shown that by dropping temperature to 0°C after a prolonged period of high temperature at 35°C–38°C, it was possible to induce hibernation in the middle of the summer phase of the endogenous clock [84].

**Figure II:**
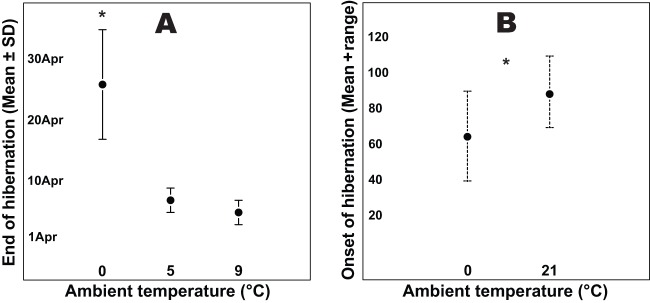
Effect of ambient temperature on hibernation phenology in squirrels. (A) Timing of spring emergence from hibernation in European ground squirrels (*Spermophilus citellus*) and (B) timing of autumn onset of hibernation in golden-mantled ground squirrels (*Citellus lateralis tescorum*) maintained under different temperatures. Drawn from (A) Nemeth et al [88] and (B) Pengelley and Fisher [84], error bars in A indicate standard deviation, and in B, data range.

### What Physiological Mechanisms Could Link Temperature and Timing?

Thermoregulation, where ambient temperature is seen as a factor regulating heat generation by the body, is a good starting point for understanding how the body perceives temperature, integrates it into the neuroendocrine system, and translates it into effector mechanisms that shape seasonal timing. We will first briefly discuss mechanisms underlying temperature perception and integration and outline four possible pathways from perceived temperature to seasonal timing.

#### A) Temperature Perception and Integration

**Figure 1:**
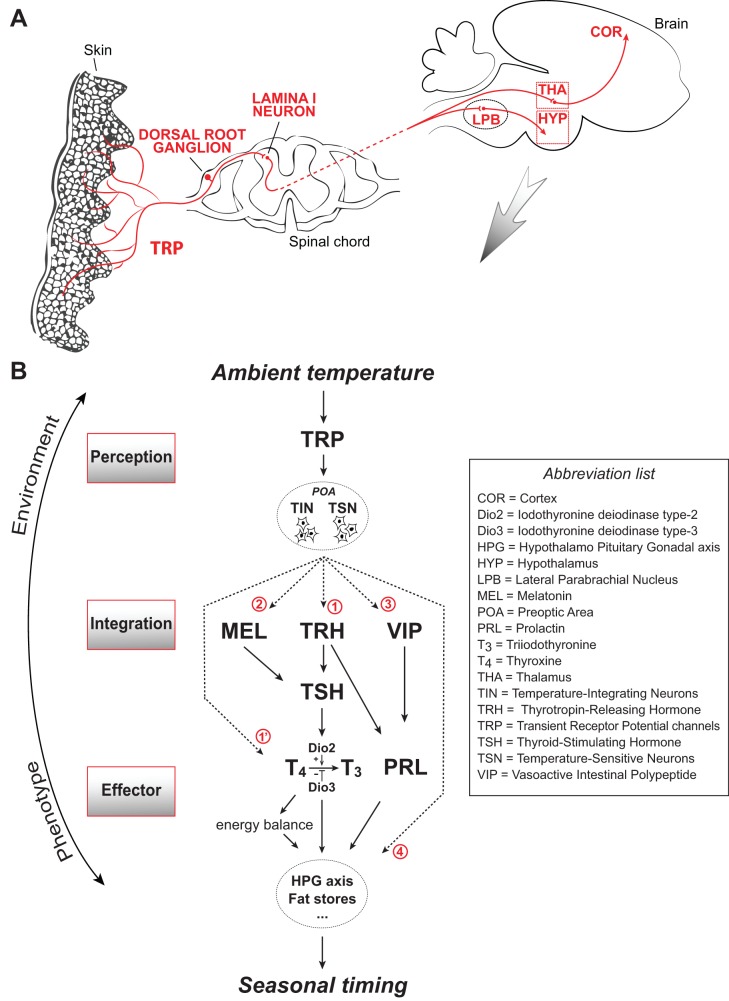
Schematic diagram depicting several hypothetical mechanisms by which ambient temperature influences seasonal timing in birds and mammals. (A) Afferent neural circuits carrying environmental temperature cues. (B) Possible mechanisms involved in the transduction of temperature cues into effector pathways that influence seasonal timing. The relevant environmental temperature cues converge towards the POA that contains both thermosensitive neurons (TSN) and neurons that integrate environmental temperature information, which we call temperature-integrating neurons (TIN). How this information is relayed to elicit physiological responses related to seasonal timing is currently a mystery. We identify four possible pathways (see numbers in red at arrowheads): 1. Via thyroid hormones: Temperature controls the expression of TRH that in turn modulates the production and release of thyroid hormones via modulation of TSH at the level of the anterior pituitary [89],[90]. Thyroid hormones can act directly on seasonal mechanisms or indirectly via the energy balance. In mammals, the sympathetic nervous system can also directly control Dio2 expression (see arrowhead 1′). 2. Via prolactin: Temperature influences PRL release via TRH or VIP. 3. Via melatonin: In mammals, but so far not in birds, MEL is known to be a powerful intermediate in the transduction of other environmental cues, such as photoperiod. In mammals, MEL modulates TSH in the pars tuberalis of the pituitary, which in turn modulates the seasonal activity of the hypothalamus [57]. 4. Within the POA: The POA integrates both internal and external temperature information, but also hosts neuropeptides that control aspects of seasonality (e.g., GnRH-I system). These two systems could be directly interconnected within the POA. These four schematic pathways would then influence mechanisms that are directly involved in the seasonal recurrence of life-cycle events such as reproduction (HPG axis), hibernation (fat stores), etc. Note that this diagram is a highly simplified representation of the mechanisms involved, which are described in more detail in the references cited in the text.

Ambient temperature is perceived by nonspecialized nerve endings in the skin (Figure 1A). Though many of these thermoreceptors, members of the transient receptor potential (TRP) superfamily [33], are activated at relatively high temperatures corresponding to the range of temperatures observed in body tissues, those activated at temperatures corresponding to “comfortable" ambient temperatures (e.g., TRPM8 (CMR1), TRPA1 (ANKTM1)) [33],[34] are potential candidates for the perception of seasonal changes in ambient temperatures.

From thermoreceptors, temperature information is transmitted via the dorsal root ganglion and lamina I neurons in the spinal chord and then splits into collateral pathways that reach the hypothalamus via the lateral parabrachial nucleus or the cerebral cortex via the thalamus (Figure 1A) [35],[36]. The first pathway is mostly involved in involuntary thermoregulatory processes, while its collateral route (the spinothalamocortical pathway) is involved in conscious temperature discrimination [36]–[38]. In the hypothalamus, the preoptic area (POA) is a critical region for temperature processing. The POA receives projections from the lateral parabrachial nucleus, but it also contains up to 30% of neurons that are intrinsically thermosensitive [39],[40], meaning that it is involved in the integration of both external and body temperatures (Figure 1B). Future research should ask whether endotherms also use these collateral pathways to time their life cycles.

#### B) Possible Effector Pathways That Can Affect Seasonal Timing

How the temperature information that reaches the brain is translated into physiological responses related to seasonal timing is probably the most important missing piece of the puzzle. In an attempt to stimulate research, we identified four pathways that we consider worthy of investigation (Figure 1B).

##### (1) Thyroid hormones

Thyroid hormones are essential intermediates in the photoperiodic organization of seasonality in endotherms [41],[42]. For example, long day lengths upregulate the enzyme (Dio2) that catalyzes the deiodination of thyroxine (T_4_) into its metabolic active form, triiodothyronine (T_3_), within the hypothalamus. T_3_ in turn stimulates reproduction by promoting the release of GnRH from neurons that originate in the POA and hypothalamus in birds [43] and mammals [44]. In several bird species, ambient temperature simultaneously affects the plasma concentrations of thyroid hormones and gonad size [45],[46], and early breeding sparrows were shown to have higher basal metabolic rates and titers of T_3_ [47]. In mammalian brown adipose tissue (BAT), adrenergic signaling through sympathetic innervation upregulates Dio2 expression, which in turn increases local T_3_ levels and activates BAT cells to produce heat in response to low ambient temperatures [48]–[51]. Thus, thyroid hormones could influence timing directly or indirectly via their effects on metabolism (Figure 1B).

##### (2) Prolactin

Elevated prolactin (PRL) concentrations, which are associated with gonadal growth, incubation, lactation, and onset of molt, have been suggested as a possible mediator of temperature on seasonality (Figure 1B). However, depending on experimental approaches and species studied, high temperatures have been shown to elevate, have no effect on, or even decrease PRL concentrations [52]–[54]. For example, in sparrows high temperature is associated with both elevated PRL titers and gonadal growth [55], while in starlings high temperature is associated with a decrease in PRL and gonadal size and an earlier onset of molt [52]. More studies on the relationship between temperature, PRL, and seasonality are therefore needed.

##### (3) Melatonin

Melatonin (MEL), produced by the pineal gland at night, is seen as the internal mirror representation of day length and is critical in coordinating seasonal timing in mammals [41],[44],[56],[57]. Birds, however, do not need MEL for responding to long day lengths (because of direct hypothalamic photosensitivity [58]). Nonetheless, MEL has been proposed to modulate sensitivity to environmental cues, and recent studies have shown that MEL influences the onset of laying and its underlying mechanisms [59]–[61]. MEL has been mostly studied in relation to photoperiod, but there is some evidence that temperature can affect both MEL fluctuations and the associated circadian rhythmicity (Figure 1B) [62],[63].

##### (4) Within the POA

In mammals and birds, the same brain areas that receive information about internal and external temperatures also express various neuropeptides that control aspects of seasonality. For example, the cell bodies of the GnRH-I neuronal system that controls reproduction in most vertebrates are present in the POA and adjacent septum [58],[64], the same brain regions that integrate information from internal and external temperature (see above). The neuron populations that sense and integrate temperature information within the POA could thus be directly connected to the neuronal systems that control the activation of the reproductive organs, without any intermediate mechanism (Figure 1B).

### How to Solve the Mystery?

To understand how ambient temperature is causally involved in seasonal timing in endotherms, we need to link the many physiological studies of temperature perception, mostly in a context of thermoregulation (Figure 1A), with studies of the mechanisms underlying seasonal timing, mostly in response to photoperiod (Figure 1B). Ecologists studying seasonal timing may play a decisive role in establishing this link, since the temperature ranges that matter in a context of seasonal timing are probably very different from those in thermoregulation, and ambient temperature acts on seasonal timing in a much more subtle way than photoperiod does [18],[65]. In addition, temperature should be viewed as an environmental signal (i.e., cue) that predicts future environmental conditions, and not only as a factor that constrains homeostasis by posing energetic challenges to the animal. As a consequence, ecologists must provide physiologists with a number of appropriate model systems: experimental set-ups with specific species kept under two specific temperature patterns, leading to a clear difference in timing between the two experimental groups.

Choosing species with appropriate ecologies is the first key task for ecologists. Avian species such as zebra finches (*Taeniopygia guttata*) are not appropriate as they use other cues (e.g., rain fall) for orchestrating their seasonal timing [66]. In mammals, where there are still few clear examples of an effect of temperature on timing, the choice of appropriate species may be more challenging. The first step might thus be to collect more data on the causal relationship between ambient temperature and seasonal timing, though the ecology of some mammalian species rules them out as potential candidates. Species with long gestation lengths are unlikely to use temperature cues for the timing of their mating as environmental conditions at the time of follicle fertilization are unlikely to predict environmental conditions months later, when the young are raised. Since species vary in their ecology, drawing general conclusions about the potential effects of a warming climate on endotherms will require the description of the relationships between temperature and phenology in a variety of model species. Within species, individuals might also differ in how they perceive, interpret, and translate temperature information into effector pathways, which has to be taken into account. For example, primates of different ages (including humans) vary in their temperature perception and thermoregulatory capacities [67]–[69].

Ecologists also need to provide physiologists with “reference experiments" in which temperature treatments are known to induce substantial differences in timing between experimental groups. We have highlighted some life-cycle stages and studies that exemplify such reference experiments (an increasing temperature affects timing of reproduction in great tits, the absolute temperature value influences molting patterns in different mammalian and bird species, etc.). Crucially, at any time it must be clear how far off animals are from their seasonal timing event so that when measurements are made it is clear which group is closer to the average timing event and which is further away. Preferably these are set-ups in which ambient temperature acts directly rather than indirectly (e.g., via food abundance) or as a constraint (e.g., low temperatures with food-deprived animals). Keeping animals exposed to artificial temperature patterns in controlled laboratory conditions fed ad libitum fulfills those requirements.

Ecologists and physiologists then need to join forces to identify the appropriate seasonal timing outputs that will be measured. A key question is whether we need to directly measure the phenotypic trait of interest (parturition date, onset of hibernation, etc.), or if we can rely on physiological proxies (e.g., hormonal concentrations, gonadal size, fat stores, etc.; see Figure 1). Using proxies has clear experimental advantages as they are usually easier to quantify than the phenotypic trait itself [70]. However, in birds, for example, the validity of the most commonly used proxies for breeding have recently been questioned [71], so should be used with care.

Once appropriate species and reference experiments have been defined, physiologists need to identify the pathways through which ambient temperature cues are integrated and translated into effector mechanisms modulating seasonal timing. How ambient temperature is perceived and integrated at the brain level has mostly been elucidated in mammals by scientists working on thermoregulation and nociception. The effector mechanisms that modulate seasonal timing have been mostly studied in birds by environmental endocrinologists and neurobiologists. Insight could also come from research conducted in very different models, even plants. Photoperiodic control of flowering in *Arabidopsis*, for example, was successfully unraveled by taking the influence of the circadian system as a starting point, leading to the discovery of crucial proteins that link circadian photoperiodism with temperature influences (e.g., [72],[73]). In vertebrates, we are currently at an earlier stage of discovery, but recently developed knowledge on mammalian circadian mechanisms in photoperiodism could be an excellent starting point, paralleling the discovery process in plant phenology. In any case, these different fields should now unite their efforts to describe the missing pieces of the puzzle. We provided four hypothetical pathways through which integrated temperature cues could mediate seasonal timing that could be tested using the reference experiments defined above.

We have argued that the link between ambient temperature perception and the effector mechanisms resides in the brain, while others have suggested that some environmental cues might affect only pathways downstream of the central nervous system, at the level of the gonads, for example [74]. If that were the case, the question of how ambient temperature regulates seasonal timing would represent an even greater mystery.

The unsolved mystery of the causal effect of temperature on seasonal timing is a major obstacle to the understanding of the biological consequences of climate change. The European Commission aims to limit the global average temperature rise to 2°C compared to the preindustrial period [75]. However, without a full understanding of how endotherms perceive and use seasonal temperature cues, the biological relevance of this 2°C upper limit cannot be properly assessed. Most of the resources and techniques necessary for revealing the link between environmental temperature and seasonal timing are already in place. Now we need to organize our diverse disciplines around common goals to solve this mystery together.

## Abbreviations

BAT: brown adipose tissue
Dio2: Iodothyronine deiodinase type-2
MEL: melatonin
POA: preoptic area
PRL: prolactin
TIN: temperature-integrating neurons
TRP: transient receptor potential
TSN: thermosensitive neurons
